# Secondary analysis of GenRED data (Genetics of Recurrent Early-Onset major Depression) using MERLIN

**DOI:** 10.1007/s00406-025-02014-y

**Published:** 2025-04-26

**Authors:** Mutaz Amin, Claudia Gragnoli

**Affiliations:** 1 Department of Biochemistry and Molecular Biology, Faculty of Medicine, Al-Neelain University, Khartoum, 11121 Sudan; 2 Division of Endocrinology, Department of Medicine, Creighton University School of Medicine, Omaha, NE 68124 USA; 3 Division of Endocrinology, Department of Medicine, Sidney Kimmel Medical College, Thomas Jefferson University, Philadelphia, PA 19107 USA; 4 Department of Public Health Sciences, Penn State College of Medicine, Hershey, PA 17033 USA; 5 Klinik für Endokrinologie, Diabetologie und Klinische Ernährung, Universitätsspital Zürich, Zürich, 8091 Switzerland; 6 Molecular Biology Laboratory, Bios Biotech Multi-Diagnostic Health Center, Rome, 00197 Italy

**Keywords:** Depression, Early-onset, Recurrent, Families, Genetic linkage, Microsatellites

## Abstract

The familial relevance of depression is a critical aspect, with evidence suggesting a substantial hereditary component. Investigating the inheritance patterns within families can provide valuable insights into the genetic underpinnings of depression. To gain a new perspective on the genetics underpinning of depression, we conducted a novel analysis of the Genetics of Recurrent Early-onset Depression (GenRED) dataset including 683 U.S. Caucasian families, each with one proband with recurrent early-onset major depression and at least one sibling with depression. Using the MERLIN tool, we identified 37 genomic markers with nominal significance linkage to early-onset recurrent depression. The findings differ from prior analyses of GenRED using a different analytical tool. Our findings highlight the variability and potential discrepancies that can arise from using different analytical tools on the same dataset. Given the chance of microsatellites undergoing duplication, mutations, and amplification errors, our two-point analysis can be more robust compared to the previous multipoint analysis. Also, one limitation is genetic admixture; studies with more homogenous ethnic groups are warranted.

**Supplementary Information:**

The online version contains supplementary material available at 10.1007/s00406-025-02014-y.

## Introduction

Depression is a pervasive mental health disorder that affects millions of individuals globally, presenting significant challenges for healthcare systems and societies [1]. Despite its prevalence, depression remains difficult to diagnose due to its multifaceted symptoms and the subjective nature of its presentation [2]. The diagnosis of depression is further challenged by its polygenic nature, where numerous genetic factors interact to influence its development [3]. Recent advances in genomics have illuminated the complex interplay of these genetic components, underscoring the importance of considering depression as a polygenic condition rather than one influenced by a single gene [4].

The familial relevance of depression is a critical aspect, with evidence suggesting a substantial hereditary component [5]. Investigating the inheritance patterns within families can provide valuable insights into the genetic underpinnings of depression [6]. Utilizing ethnically homogenous datasets, which minimize genetic and environmental variability, can more effectively dissect the hereditary aspects and identify potential genetic markers associated with depression [6].

To gain clearer insight into these hereditary aspects and identify genetic markers associated with depression, we used a novel approach to analyze microsatellite DNA markers in a large, preexisting dataset of families with a history of major depression. In this study, we analyzed 374 microsatellite DNA markers in 683 families, each with one proband with recurrent early-onset major depression and with at least one sibling with depression and identified 37 genomic markers with nominal significance linkage to early-onset recurrent depression.

## Materials and methods

The GenRED study represents a significant effort in the field of psychiatric genetics, striving to elucidate the hereditary components of a debilitating and common mental health disorder [7]. This large study focused on identifying genetic factors associated with recurrent early-onset major depression. It examined microsatellite DNA markers across the genome of 656 families [7] using the ALLEGRO software [8], a primary multipoint allele-sharing linkage analysis powerful in detecting regions of the genome potentially harboring susceptibility loci for complex traits.

We re-analyzed the GenRED dataset (Genetics of Recurrent Early-onset Depression) using MERLIN tool (Multipoint Engine for Rapid Likelihood Inference) [9]. MERLIN employs various algorithms to calculate test statistics, including but not limited to LOD scores, non-parametric and parametric, linkage-disequilibrium model-based LOD scores, infer haplotype, and detect errors, enabling researchers to identify regions of the genome that co-segregate with a trait of interest. Its robust capabilities and flexibility make it a valuable resource for mapping genetic loci associated with various diseases and traits [9].

Detailed methods for the initial genetics analysis of this dataset are provided in GenRED [7]. But briefly, 374 microsatellite DNA markers were analyzed in 683 families, each with one proband with recurrent early-onset major depression and with at least one sibling with depression. Probands were recruited from 6 major U.S. sites [10]. Cases with recurrent early-onset major depressive disorder were required to have had at least two-lifetime major depressive episodes (extending beyond age 18) or a single episode lasting 3 or more years, accompanied by significant function impairment. The age of onset had to be before 31, with a high level of diagnostic confidence. Blood specimens were collected from interviewed subjects, their available parents, and, if fewer than two parents were available, up to two siblings without a known major depressive disorder, as reported in the Family Interview for Genetic Studies [10].

We re-analyzed the GenRED familial data of the 683 U.S.-Caucasian families, by conducting a two-point non-parametric allele-sharing analysis using the MERLIN software. The U.S.-Caucasian 683 families we studied had the following structure: 6,021 individuals (2,051 founders, 3,970 non-founders), including 3,359 females and 2,662 males, with an average familial size of 8.82 (4 to 48), and an average family size distribution of 4 (22.8%), 5 (16.1%), and 7 (10.4%). The familial average generation was 2.49 (2 to 5), with a generation distribution of 2 (62.2%), 3 (26.6%), and 4 (11.0%).

MERLIN facilitates genetic linkage analysis by efficiently handling large datasets and complex pedigrees. We performed nonparametric linkage (NPL) both across the whole dataset (NPL-all) as well as between pairs (NPL-pair). P-value ≤ 0.05 was considered statistically significant at the nominal level.

## Results and discussion

**Fig. 1:**
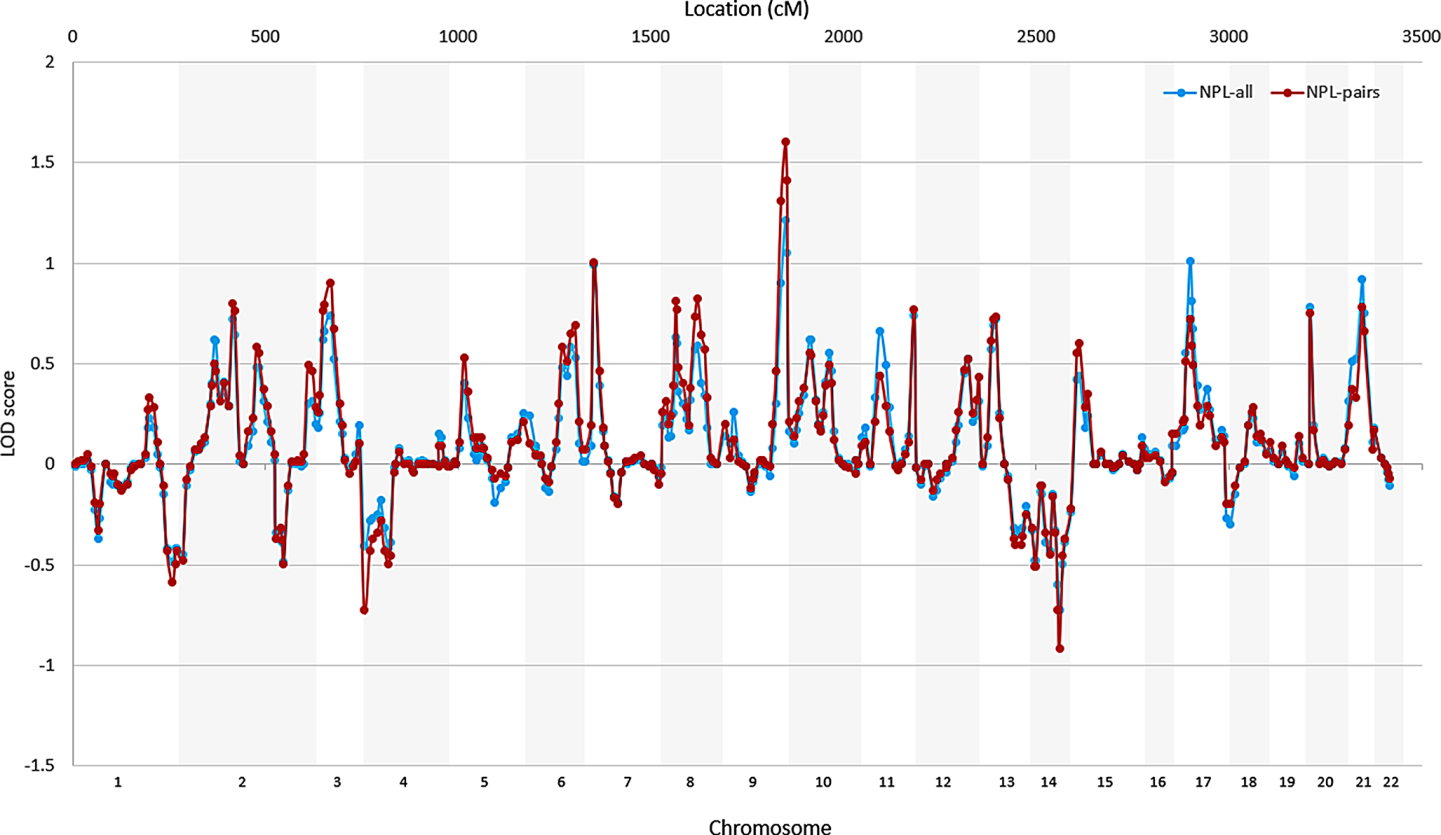
Chromosomal locations of markers with nominal significance linkage (*P* ≤ 0.05) in either NPL-all or NPL-pair analyses to recurrent early-onset depression using MERLIN

**Fig. 2:**
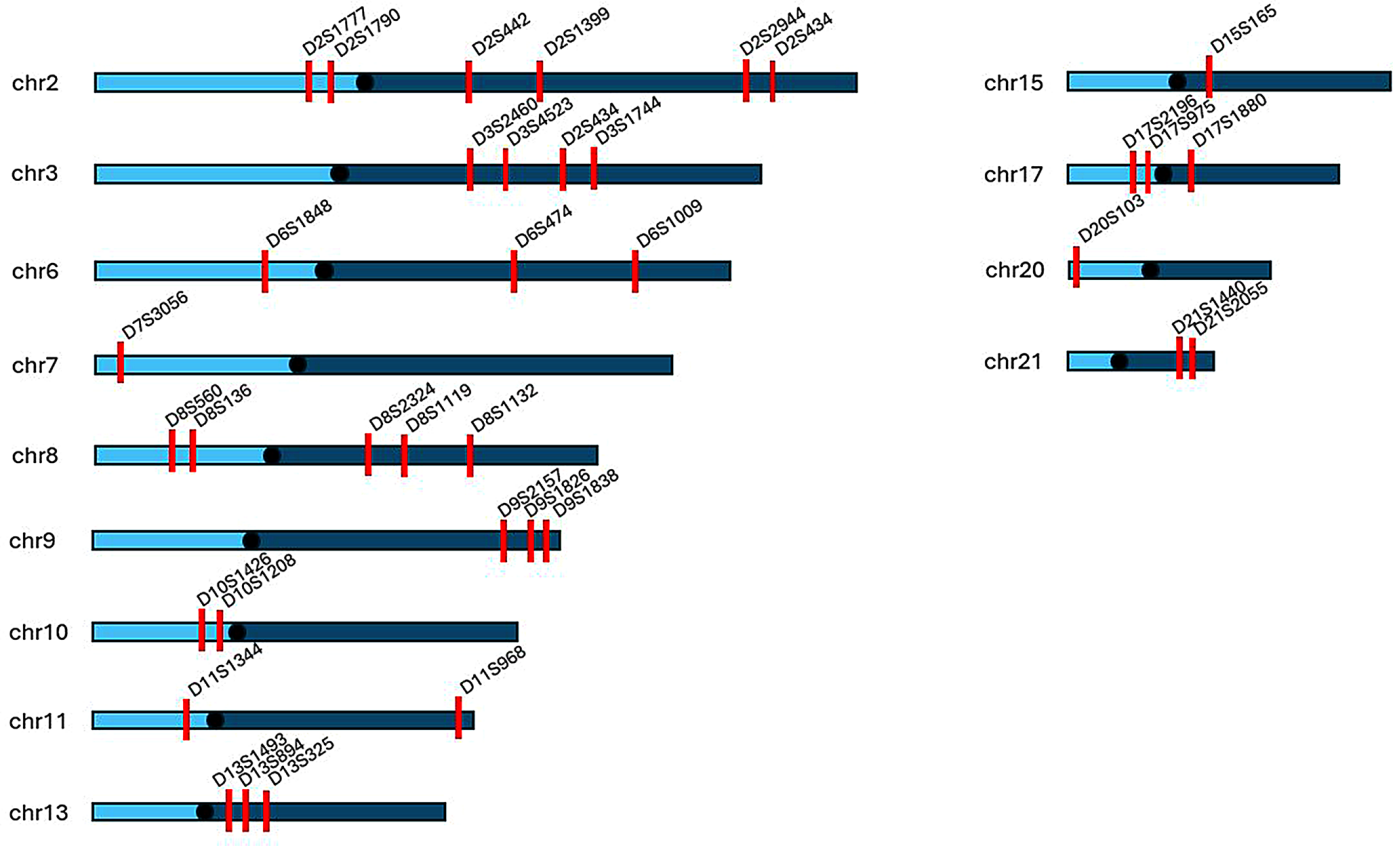
Genome-scan non-parametric linkage results (NPL) of all (NPL-all) and pair-wise analysis (NPL-pair) (using MERLIN) of cases with recurrent early-onset major depressive disorder

We detected 37 genome-wide nominal significance linkages to early-onset recurrent depression on chromosomes 2, 3, 6–11, 13, 15, 17, 20 and 21 (*P* ≤ 0.05) (Fig. 1). While these findings do not meet the conventional genome-wide significance threshold (*P* ≤ 5 × 10⁻⁸), they highlight potential loci that warrant further investigation. The LOD score of all tested markers under NPL-all and NPL-pair is shown in Fig. 2, and markers nominally significant in either NPL-all or NPL-pair are shown in Supplementary Table 1. Specifically, 29 markers showed significance in NPL-all, while 31 markers were significant following pairwise analysis (NPL-pair). Among these, 23 markers were significant in both NPL-all and NPL-pair analyses (Fig. 2). The NPL-pair analysis demonstrated greater power than the NPL-all, primarily due to the inclusion of affected pairs, while the latter analysis had to account for the uncertainty associated with relatives who were not yet affected.

In both the NPL-all and the NPL-pair analyses, the maximum LOD scores were observed for two markers in chromosome 9: D9S1826 and D9S1838, with LOD scores of 1.21 (*P* = 0.009) and 1.05 (*P* = 0.014) in NPL-all, respectively, and with LOD scores of 1.60 (*P* = 0.003) and 1.41 (*P* = 0.005) for NPL-pair, respectively (Fig. 2) (Supplementary Table 1).

A Chi-square goodness of fit (GOF) test was performed to determine if the percentages of markers in the top 10% were randomly distributed across chromosomes. The GOF test yielded a P-value of 0.02 for the NPL-all statistics, indicating that some chromosomes were overrepresented in the top 10%. The chromosomes with the highest representation in the top 10% for NPL-all were chromosomes 2, 3, 8, 9, 10, 11, 13, 17, and 20. Similarly, the GOF test resulted in a P-value of 0.03 for the NPL-pair analysis, again suggesting a non-random distribution of top markers across chromosomes. The most represented in the top 10% for NPL-pair were chromosomes 2, 3, 6, 8, 9, 10, 11, 13, and 17.

These findings highlight specific chromosomes that could be of interest for follow-up studies, particularly those consistently represented in the top 10% of markers for both NPL-all and NPL-pair analyses. The count of chromosome markers in the top 10% is shown in Table 1. The top 10 markers are shown in Table 2.

**Table 1:** Top 10% genetic markers identified in goodness of fit (GOF) analysis

Chromosome	Count of Chromosome Markers in Top 10%	Proportion in Top 10%	ChiSquare GOF Test
2	4	10.81	3.20
3	4	10.81	3.20
8	4	10.81	3.20
17	4	10.81	3.20
21	4	10.81	3.20
9	3	8.11	1.03
10	3	8.11	1.03
11	3	8.11	1.03
13	3	8.11	1.03

**Table 2:** Top 10 genetic markers of the NPL-all and NPL-pairs analyses

Chr	Marker	Chr Position (cM)	Genome Position	NPL-all-LOD	p-value-NPL-all	NPL-pairs-LOD	p-value-NPL-pairs
9	D9S1826	157.73	135561126	1.21	0.009*	1.6	0.003*
9	D9S1838	161.73	137743964	1.05	0.014*	1.41	0.005*
17	D17S2196	47.32	17364299	1.01	0.02*	0.72	0.03*
7	D7S3056	8.69	4455712	0.99	0.02*	1	0.02*
21	D21S1440	45.39	37769324	0.92	0.02*	0.78	0.03*
9	D9S2157	146.54	133161774	0.9	0.02*	1.31	0.007*
17	D17S975	52.78	29786105	0.81	0.03*	0.59	0.05*
20	D20S103	2.52	580337	0.78	0.03*	0.75	0.03*
21	D21S2055	49.25	39821167	0.75	0.03*	0.66	0.04*
3	D3S1764	145.53	139480006	0.74	0.03*	0.9	0.02*

The nominally significant microsatellite markers we report are near 37 genes, none of which have been previously reported in association with depression (Supplementary Table 1). Notably, 13 of these markers have been linked to other psychiatric and metabolic disorders, including schizophrenia (D9S1838 [11]), substance abuse (D9S1826 [12], bipolar disorder (D13S1493 [13]) and metabolic syndrome (D6S1009) [14]. Several nearby genes are components of relevant pathways and/or diseases, such as neuronal function (associated with *IGSF9B* [15], *GFRA2* [16] and *TNFSF11* [17]), cell cycle control (associated with *CSNK2A1* [18]) proteasome activity (associated with *CUL2* [19] and *PSMD9* [20]), the latter reported in linkage to depression [21], Wnt-signalling (associated with *TCF7L2* [22] and *TRABD2A* [23]), also linked to depression [24] (which could be linked to infection [25]) molecular transport (associated with *SLC7A13* [26]), fibromyalgia (associated with *NT5M* and *MED9*), which may also indicate a predisposition to infections [27]), and metabolic disorders associated with *NHEG1* [14] and *PCP4*, specifically under low protein diet [28]). Of note, a low protein and calorie diet is predisposing to depression [29]. (Supplementary Table 1). Notably, D2S2944 has been previously linked to recurrent early-onset depression [30]. Our findings support the polygenic nature of major depressive disorder, aligning with previous evidence that no single genetic locus has a dominant effect on overall risk [31]. Instead, multiple loci, potentially including those identified in our study, may contribute to disease susceptibility [5]. To assess the consistency of our findings with existing genetic studies, we compared our results to the Psychiatric Genome Consortium (PGC) major depression GWAS [32] and found that one gene, *IGSF9B*, which contains two reported SNPs in the PGC dataset, is in close proximity to the microsatellite marker D11S968 identified in our study. This overlap suggests a potential relevance of this locus in major depression and highlights the need for further investigation using high-resolution genomic approaches.

The broader implications of our findings suggest involvement in multiple biological pathways. *SPAG16* (Sperm associated antigen 16), previously identified as genome-wide significant in type 2 diabetes (T2D) [33], has been linked to depression via its role in semen’s antidepressive activity [34]. This supports a genetic connection between depression and metabolic disorders, as observed in previous studies [35–38]. Additionally, *SPAG16* is associated with rheumatoid arthritis [39] and multiple sclerosis [40], reinforcing potential links between depression and autoimmune diseases. Markers linked to inflammatory pathways and gastrointestinal disorders, such as Crohn’s disease, further highlight the gut-brain axis’s role in depression [41]. Another notable gene, *OBP2A*, related to the olfactory function, has been implicated in both T2D [33, 42], and depression [43], underscoring shared genetic mechanisms across these conditions.

These findings emphasize the complex genetic architecture of major depression and suggest potential cross-links with other metabolic, immune-related disorders, and psychiatric diseases, the latter also comorbid with immune, metabolic, and mental disorders [44–48]. Future studies using high-resolution genomic approaches will be essential to also refine these comorbid disorder’s associations and further elucidate their biological significance.

The biological relevance of the identified loci is further supported by the pathways in which these genes are involved. Several of these pathways are directly implicated in depression, neuroinflammation, and neurotransmission. For instance, axon guidance and nervous system development are fundamental for synaptic connectivity and plasticity [49], both of which are altered in depression [49–51]. Disruptions in these processes can contribute to impaired neuronal communication and affect mood regulation [51, 52].

Moreover, pathways related to metabolism, including lipid metabolism and protein metabolism, have been increasingly recognized in the pathophysiology of depression [53, 54]. Dysregulated lipid metabolism has been linked to altered membrane composition, affecting neurotransmitter receptor function and signalling [55]. Similarly, protein metabolism, particularly post-translational modifications, may influence neuroinflammatory responses and synaptic function [56].

A more detailed list of pathways containing two or more components is provided in Supplementary Table 2. These findings highlight the complex interplay between neurodevelopmental processes, immune signalling, metabolic regulation, and transcriptional control in the biological underpinnings of depression.

The prior GedRED analysis was rigorous, and it uncovered several genomic loci and markers underpinning the genetics of recurrent early-onset major depression, providing insights into the biological pathways involved and potentially guiding future therapeutic interventions [7].

The findings from this study diverge from the previous analysis conducted using the ALLEGRO tool. Specifically, the top markers previously associated with early recurrent depression (D15S652 and D15S816 on chromosome 15) in GenRED are insignificant in our current study. This highlights the potential differences between different genome-wide analytic tools. ALLEGRO and MERLIN are both powerful tools used for genome-wide linkage studies, each with its unique strengths and outputs [57]. ALLEGRO, known for its efficiency in handling childhood-onset diseases, employs multipoint linkage analysis and is particularly effective in calculating allele-sharing statistics and LOD scores [57]. MERLIN, on the other hand, offers a comprehensive suite of functions (e.g., error detection, haplotype inference, and permutation testing) [9]. One notable difference is MERLIN’s ability to detect and correct genotyping errors, enhancing its linkage results’ accuracy. Additionally, MERLIN can handle more complex family structures and provides detailed haplotype information, which can be crucial for fine-mapping studies [9]To provide a clearer comparison, we summarize the key differences between ALLEGRO and MERLIN [58, 59] in Table 3.

**Table 3:** Summary of the key differences in analytic performance between MERLIN and ALLEGRO

Feature	Allegro	Merlin
Analysis Type	Multipoint linkage analysis	Multipoint linkage analysis
Parametric LOD Scores	Yes	Yes
Non-Parametric Analysis	Yes	Yes
Haplotype Inference	Limited	Yes
Handling of Dense Marker Maps	Moderate	Efficient
Computational Speed	Fast	Faster

Our findings highlight the variability and potential discrepancies that can arise from using different analytical tools on the same dataset [60]. Using Caucasian families from different geographic sites in the USA might implicate genetic allelic differences and admixture hidden effects, impairing the results. Future research should consider integrating multiple analytical approaches to provide a more comprehensive understanding of the genetic underpinnings of complex traits such as early-onset depression. Furthermore, the use of even more homogenous, non-admixed, or inbred populations will help detect genetic loci contributing to complex heterogeneous disorders such as depression. A notable limitation of this study is the inherent susceptibility of microsatellites to mutations, duplications, and other genetic variations [61] which can introduce genotyping errors and impact linkage results. Microsatellite instability (MSI), in particular, can lead to allele shifts, either in the germ line or somatic cell, that obscure the true inheritance patterns, potentially inflating or deflating linkage signals. Such instability may be more pronounced in affected individuals, leading to an overrepresentation of certain marker allelic mutation in the dataset and complicating their interpretation as putative disease loci and overall impairing linkage results [62]. Furthermore, genotyping errors arising from slippage events (i.e., erroneous nucleotide duplications by the DNA polymerase during DNA replication) or miscalling of alleles can introduce noise into the analysis, potentially leading to false-positive or false-negative linkage findings. In affected individuals, DNA and microsatellite mutation rates are increased due to factors such as oxidative stress and inflammation leading to higher mutations and reduced DNA repair function [63]. Additionally, if a microsatellite marker linked to the disease undergoes rearrangements across generations and families, it could further complicate the analysis and interpretation of results. A potential consideration is that the microsatellites might have further impaired the multipoint locus power analysis in the previous study; this might explain why markers detected by the two-point analysis were not reported in the previous multipoint analysis study.

To overcome these challenges, follow-up studies should incorporate single-nucleotide polymorphism (SNP) arrays or next-generation sequencing (NGS) for fine-mapping these loci. These high-resolution approaches can provide more precise genotypic data, reduce error rates associated with microsatellites, and facilitate the identification of causal variants within linked regions. Integrating such technologies will enhance the accuracy of disease locus mapping and improve the overall reliability of genetic linkage studies.

## Electronic supplementary material

Below is the link to the electronic supplementary material.

Supplementary Material 1

Supplementary Material 2

## Data Availability

The data used were accessed through the NIMH database.
